# Suppressing kidney angiotensinogen in blood pressure regulation

**DOI:** 10.14814/phy2.12704

**Published:** 2016-02-04

**Authors:** Takamitsu Saigusa

**Affiliations:** ^1^ Division of NephrologyMedical University of South CarolinaCharlestonSouth Carolina

The classical renin–angiotensin system (RAS) is activated by renin that is secreted from the juxtaglomerular cells of the afferent arteriole, which cleaves angiotensinogen (Agt), synthesized and released by the liver. This leads to the generation of a potent biologically active peptide, angiotensin II (AngII), which contributes to blood pressure regulation and sodium homeostasis. Recent studies have gone beyond the classical RAS pathway, to suggest that local production of RAS, particularly in the kidney, is essential for salt and water homeostasis and blood pressure regulation. Current evidence indicates that all RAS components necessary to generate Ang II are locally produced in most nephron segments (Rohrwasser et al. 1999), and can do so, independently from the classical RAS pathway (Kobori et al. 2007). Inappropriate activation of these components may lead to the development of hypertension or chronic kidney disease and thus, it is important to understand the regulation of the local kidney RAS system.

Agt in the kidney is primarily synthesized in the proximal tubule (PT) (Ding et al. 1997). For example, transgenic mice that overexpress human Agt, under a human Agt promoter, have high levels of human Agt selectively in the PT (Ding et al. 1997). Upregulation of Agt in the PT appears to be physiologically significant, since mice overexpressing Agt, specifically in the PT, develop salt‐sensitive hypertension (Ying et al. 2012). Agt synthesized in the PT is released into the tubular lumen, resulting in generation of AngII. Interestingly, AngII appears to stimulate further synthesis of Agt in the PT, perpetuating intrarenal RAS activity (Kobori et al. 2007). Therefore, Agt in the urine maybe a biomarker reflecting intrarenal RAS activity (Kobori et al. 2002), while targeting Agt in proximal tubules may have an important implication in blood pressure regulation (Ramkumar and Kohan 2013).

Although Agt is synthesized in the PT, the major source of proximal tubular Agt, which leads to the generation of kidney AngII, is controversial. For example, in vivo visualization of glomerular permeability to Agt using two‐photon microscopy in normal mice and proteinuric rats demonstrated that systemic administration of uncleavable fluorescent‐labeled human Agt was minimally detected in the urine, indicating that Agt primarily originates from the tubules rather than from glomerular filtration (Nakano et al. 2012). On the other hand, Matsusaka and coworkers demonstrated that although there were no changes in urinary Agt levels, kidney Agt and AngII levels were decreased in liver‐specific Agt knockout (KO) mice compared to kidney‐specific Agt KO mice (Matsusaka et al. 2012). Notably, these liver‐specific Agt KO mice were hypotensive, but blood pressure in kidney‐specific KO mice were normal, suggesting Agt in the PT primarily derives from the liver and contributes to local AngII generation and systemic blood pressure. However, there is some uncertainty as to whether the kidney androgen‐regulated protein promoter, used to specifically KO Agt in the kidney, is capable of fully deleting Agt in the proximal tubules. This is especially true since urinary Agt levels were not suppressed in their study (Matsusaka et al. 2012). In addition, whether inhibiting Agt specifically in the PT effects blood pressure under conditions of high levels of circulating AngII is currently unknown.

In this current issue, Ramkumar et al. (2016) created a nephron‐wide Agt KO mice by cross breeding a Agt floxed allele mouse (Matsusaka et al. 2012) to a mouse with Pax8‐rtTA and LC‐1 transgene. This tetracycline/doxycycline‐inducible mouse is under control of the mouse *Pax8* promoter with high expression of the reverse tetracycline transactivator (rtTA) in all segments of the nephron (Traykova‐Brauch et al. 2008). LC‐1 is a transgene which encodes tetracycline/doxycycline‐inducible cre and luciferase (Schönig et al. 2002). Administering doxycycline triggers rtTA to bind to LC‐1 transgene, leading to expression of luciferase and cre recombinase in the renal tubular cells, which enables Agt to be specifically deleted in renal epithelial cells. These mice indeed show reduced expression of Agt mRNA and protein levels in the proximal tubules, decreased plasma/urinary Agt levels, and increased plasma renin concentration. These findings were true regardless of different salt intakes. Most importantly, systolic blood pressure was significantly lower compared to control mice at baseline and during normal, high‐, or low‐salt diet. Notably, blood pressure and urinary Agt level in the knockout mice were markedly reduced compared to control when given a low‐salt diet. Chronic AngII infusion significantly increased blood pressure in control mice, but was attenuated in Agt KO mice. The expression of Na^+^/H^+^ exchanger (NHE3) was lower in the Agt KO mice compared to controls. These results indicate that suppressing Agt in the nephron plays a role in lowering systemic blood pressure regardless of different salt intakes and even with high‐circulating AngII levels.

However, it should be pointed out that this mouse model lacks site‐specific deletion of Agt. Not only was Agt deleted in the nephron, but it was also decreased in liver and in plasma, which apparently can be seen in Pax8‐rtTA‐based gene deletion, possibly from targeting periportal hepatocytes (Traykova‐Brauch et al. 2008). From this perspective, the reduction in plasma Agt may have been derived from Agt synthesized from the liver, which makes it difficult to distinguish whether the BP lowering effect is truly from the nephron versus a systemic effect. While a mouse model with site‐specific gene deletion of the nephron would be ideal, we must acknowledge that Cre‐expressing mouse models have limitations.

In summary, the findings of Ramkumar et al. (2016) demonstrate a potential role of nephron‐derived Agt in effecting systemic blood pressure under high systemic AngII levels. A caveat to this Pax8‐rtTA Agt mice is the unexpected suppression of liver and plasma Agt which may have played a role in altering blood pressure in this mouse model. Nonetheless, their results show that urine Agt is primarily derived from the nephron, and targeting Agt in the kidney, at least under high‐circulating AngII states, may have a role in suppressing intrarenal RAS and possibly hypertension. Although there is no known Agt inhibitor approved for humans, an antisense oligonucleotide targeting Agt synthesis is being tested in polycystic kidney disease mice; the results suggest that it may be a potent intrarenal RAS inhibitor (Saigusa et al. 2016). As we gain more evidence investigating the role of Agt in blood pressure regulation, targeting Agt may become clinically relevant and essential therapeutic tool for the treatment of hypertension.

## Conflict of Interest

None declared.
